# Combining development, capacity building and responsible innovation in GCRF‐funded medical technology research

**DOI:** 10.1111/dewb.12340

**Published:** 2022-03-26

**Authors:** Louise Bezuidenhout, Julian Stirling, Valerian L. Sanga, Paul T. Nyakyi, Grace A. Mwakajinga, Richard Bowman

**Keywords:** development, ethics, GCRF, LMIC, medical technologies

## Abstract

Development‐oriented funding schemes such as the UK Global Challenges Research Fund (GCRF) have opened up opportunities for collaborations between low‐middle income countries (LMICs) and high‐income country (HIC) researchers. In particular, funding for medical technology research has seen a rise in previously under‐represented disciplines such as physics and engineering. These collaborations have considerable potential to advance healthcare in LMICs, yet can pose challenges experienced to researchers undertaking these collaborations. Key challenges include a lack of tradition of HIC/LMIC collaborations within participating departments, lack of experience with development agendas, designing contextually‐appropriate technologies and ensuring long‐term viability of research outputs. This paper reflects on these key challenges, using the experiences of the authors on the Open Laboratory Instrumentation (OLI) project as a focalizing lens. This project was a GCRF‐funded collaboration between physicists in the UK and engineers in Tanzania to develop an open‐source, 3D‐printed, fully‐automated laboratory microscope. The paper highlights key ethics lessons learnt.

## 1 AN EVOLVING LANDSCAPE OF HEALTH‐RELATED RESEARCH

Recent decades have seen a rapid expansion in healthcare technologies. These include artificial intelligence (AI), virtual/augmented reality (VR/AR), 3D printing, robotics and nanotechnology. These digital technologies are widely expected not only to transform and expand current healthcare provision, but also to transform unsustainable healthcare systems into sustainable ones, lower the cost of faster and more effective healthcare solutions, and improve relationships between medical professionals and patients.

The expansion of healthcare technologies has seen a rise in the prominence of disciplines such as engineering, physics and computer science in the health landscape. Advances in information technologies and artificial intelligence are combining with advances in the biological sciences; including genetics, reproductive technologies, neuroscience, synthetic biology; as well as advances in the physical sciences to offer unprecedented opportunities for advancement. Academic and commercial researchers from these disciplines are not only increasingly involved in collaborative healthcare‐focused projects, but becoming lead investigators of health‐focused grants in their own right.

### 1.1 Ethics and digital diagnostics

The advances in healthcare technologies has occurred against the backdrop of the “4^th^ Industrial revolution (4^th^ IR).” The World Economic Forum describes the 4^th^ IR as “the advent of “cyber‐physical systems” involving entirely new capabilities for people and machines.”^1^ The 4^th^ IR represents entirely new ways in which technology becomes embedded within societies and even our human bodies. The 4^th^ IR raises a wide range of ethics concerns relating to the design and distribution of technologies.^2^

In response to these challenges, there is growing global recognition of the need for governance and for educating researchers and innovators to responsibly produce technology that benefits society. Highly influential frameworks, such as Responsible Research and Innovation (RRI) have been introduced. RRI recognises that the technologies of the 4^th^ IR are changing the way we live and work.^3^ RRI was defined by von Schomberg as: “a transparent, interactive process by which societal actors and innovators become mutually responsive to each other with a view on the (ethical) acceptability, sustainability and societal desirability of the innovation process and its marketable products (in order to allow a proper embedding of scientific and technological advances in our society).”^4^

Within the RRI framework practices of societal co‐production and anticipatory harm mitigation are key. Through these practices RRI aims to foster the “design of inclusive and sustainable research and innovation.”^5^ Key to the RRI approach are the stages of reflection. The model for these stages was initially proposed by Stilgoe, Owen and Macnaghten in 2013, and comprises four interlinking stages: anticipation, reflexivity, inclusion and responsiveness.^6^ Later adaptations of the RRI model, such as that proposed by the EPSRC, adapted these stages to anticipation, reflection, engagement and acting (“AREA”). Successful implementation of the RRI requires considerable investment from all stakeholders. Not only to research teams need to engage in critically reflexive dialogue, but also foster two‐way dialogue with non‐academic stakeholders. This dialogue focuses on opening up the research visions, impacts and questioning to broader deliberation, dialogue, engagement and debate in an inclusive way.^7^

Like many ethical frameworks, the RRI model is not prescriptive of how the AREA practices are implemented within a specific project. Indeed, RRI continues to be a largely top‐down approach guided by standardized principles.^8^ Nonetheless, there are a growing number of case studies demonstrate the heterogeneity of the transition from model to practice.^9^ To date, however, RRI has mainly been applied to high‐technology innovation in the Global North. As mentioned by Hartley et al, “there is a significant gap in the literature relating to the application of RI in low technology, Global South contexts,^10^ or outside Western liberal democratic contexts.”^11^^,^ 12

The focus on high‐technology and high‐income countries (HICs) potentially poses problems for researchers entering into collaborative health technology research with low/middle‐income country (LMIC) researchers. It not only means that the language and examples fit the social background of researchers working in HICs, but also that they presume the existence of institutional and national regulatory infrastructures, as well associated resources such as funding, educational support and advice. The difficulties of engaging with ethics discussions are further compounded by what Macnaghten et al describe as “ideological coercion.”^13^ By this they mean the risk of imposing a concept or normative model on to a LMIC context with “little regard for its context and for the assumptions that RI as a Northern political artifact brings (e.g. in terms of culture, politics, economy, demographics, governance and power structures, institutional arrangements, science and society relationships).”^14^ This could result in HIC researchers unwittingly engaging in neo‐colonialist practices that could unintentionally “reproduce or reinforce relations of dependence that are far from emancipatory for the global South.”^15^

Current ethics of technology frameworks, such as RRI, thus present potential challenges for health care technology projects. In particular, projects focusing on developing technologies for use in LMICs are under‐supported by the current literature and training. The paucity of case studies detailing the roll‐out of RRI‐guided technology development in LMICs offers little in the way of guidance for researchers entering into this realm. Moreover, for researchers entering into these projects from natural science disciplines, the lack of connection between RRI and other ethics guidance with the longstanding traditions of (bio)medical ethics can mean that key learning opportunities are overlooked.

## 2 COMBINING RESPONSIBLE INNOVATION WITH A DEVELOPMENT AGENDA

The 4^th^ IR relies on responsible, robust and responsive research systems. In contrast to most HICs, many LMIC research institutions, systems and infrastructures face many historic challenges that continue to hamper efficient growth. These include inherited colonial higher education systems, parachute research^16^ and an historic lack of capacity building investment, limited resource investment in research infrastructures and systems and under‐representation of LMIC academics from global research communities.^17^ Continental and national development strategies are thus intimately bound to research capacity building activities on the continent.^18^

### 2.1 The Global Challenges Research Fund

The field of medical technology research has recently become a priority area for many (inter)national funders. The structure of this funding has varied from traditional research grants to philanthropic/commercial investment. In 2015 UK Research and Innovation (UKRI) launched the Global Challenges Research Fund (GCRF). Unlike previous funding, the GCRF formed part of the UK's Official Development Assistance (ODA) commitment, which is monitored by the Organisation for Economic Cooperation and Development (OECD). Drawing down £1.5 billion of Official Development Assistance (ODA) funding over 2016‐2021, the GCRF was envisioned as contributing to realisation of the ambitions of the UK aid strategy and to make progress on the global effort to address the UN Sustainable Development Goals^19^ (SDGs).^20^ The fund constituted a significant investment of ODA money in research.

GCRF calls for “meaningful and equitable” research partnerships between UK‐based academics and partners in the Global South.^21^ The GCRF thus links to a growing discourse outlining both the need for, and the establishment of, equitable partnerships in research between LMICs and HICs. Frameworks for equitable partnership have emerged over in recent years that identify key elements such as co‐creation, communication, commitment and continuous review. These elements are supported by ethical values such as fairness, respect, care and honesty.^22^

The GCRF was designed as a UK Government initiative “to ensure UK science takes the lead in addressing the problems faced by developing countries, whilst developing [the] ability to deliver cutting‐edge research.”^23^ The GCRF thus represented a new approach to research funding combining research project funding with priorities from the development agenda and societally responsive research commitments. Indeed, the GCRF had a number of key objectives, including to:

- promote challenge‐led disciplinary and interdisciplinary research, including the participation of researchers who may not previously have considered the applicability of their work to development issues
- strengthen capacity for research, innovation and knowledge exchange in the UK and developing countries through partnership with excellent UK research and researchers
- provide an agile response to emergencies where there is an urgent research need^24^

The GCRF funding scheme received considerable support for its focus on “blue skies” research on key issues such as secure and resilient food systems, sustainable health and well‐being, inclusive and equitable education, clean air/water/sanitation and sustainable energy. Its potential was also recognized as an important contributor to research equity, and a key means of diversifying the research undertaken as North‐South collaborations. In particular, it facilitated the inclusion of research outside of the well‐established medical and healthcare collaborations and networks to include other fields such as engineering research.

The expected outcome of the funding scheme was to facilitate the deployment of UK research excellence to address the most “significant and complex problems faced by the developing world, while at the same time strengthening research capability in developing countries.”^25^ In order to do this, it explicitly highlighted the need for meaningful and equitable relationships between the UK and developing country partners to identify context‐appropriate solutions to existing problems. The inclusion of the development agenda into research funding introduced a new set of ethical commitments for researchers receiving grants from this fund.

### 2.2 Mixing research and development agendas

The GCRF makes an important contribution to the establishment of a wide‐ranging research agenda covering a range of development issues. Nonetheless, to be properly realised, GCRF‐funded academics to demonstrate specific skills and experience of working effectively with colleagues and partners in the Global South.^26^ For many academics, this represents a departure from their prior research experiences, and requires a shift from more traditional roles of academics as passive observers of development practice. Instead, they are increasingly expected to take an active role in contexts where networks of actors with varying levels of power and capacity are competing and collaborating with one another to bring about change for specific groups of people.^27^ In order to be effectively supported in these activities, it is important that academics have access to training on how best to run development‐focused activities or research projects.

Key skills required for robust development agendas include getting buy‐in from local communities, working with both academic and non‐academic partners, coordinating and managing relationships between delivery partners. Some of these challenges are detailed in Table 1 below. Successful development activities also require systems to monitor and evaluate the success of these development agendas. These requirements also reflect the recognized challenges of effective equitable research partnerships. Implementing frameworks for equitable research partnership are recognized to require expertise, time and resources to implement. Indeed, a range of papers have highlighted the critical importance of leadership, resolution and resourcing in the successful implementation of equitable partnership relations.^28^

**Table 1:** Key components of development‐focused research and possible challenges for GCRF grant holders. Adapted from Datta 2018

Key requirements for successful development‐focused research	Challenges for GCRF grant holders
To engage with counterparts in the Global South	How to deal with capacity development, cross‐cultural communication, understanding research contexts and negotiating time pressures of grants
To engage across disciplinary boundaries	How to develop shared conceptual frameworks and develop the necessary trust relationships
To engage with other stakeholders such as policymakers and practitioners	How to engage instead of inform/disseminate
To communicate risk and uncertainty to policymakers and practitioners	How to include different perspectives through gender, cultural and other factors
To manage, reflect on, and learn about the effects and impacts they are having (or not) on development processes in the Global South	How to manage impacts without being trained in “stakeholder engagement”, “adaptive management,” or the “theory of change”

Interestingly, these challenges have been recognized by the UKRI since the GCRF inception.^29^ Nonetheless, the responsibility for supporting individual academics has been largely devolved to individual institutions rather than as a centralized support from the funding body.

It is important to recognize that the GCRF scheme has also expanding funding to disciplines that have not traditionally been as involved in HIC/LMIC collaborations. Disciplines such as health and education, that have longstanding debates on the ethics of HIC/LMIC research, equitable partnerships and collaboration. In contrast, similar debates within disciplines such as physics and engineering are only now emerging. Thus, while ethics and physics projects are increasingly funded under the GCRF, researchers do not benefit from the extensive, disciplinarily‐focused guidance available to their health and social sciences colleagues. This means that researchers from these disciplines are entering into new forms of LMIC/HIC collaborations in new, technology‐focused areas that open both new opportunities for development‐focused research without disciplinarily‐specific ethics guidance on the complex issues elaborated above.

## 3 OLI AS A CASE STUDY

Receiving GCRF funding for health technology development is thus more complicated than simply delivering excellence in research. The funding places ethical commitments on researchers in relation to responsible innovation and development/capacity building. In addition, it places expectations on researchers to engage in ethical‐focused research activities such as equitable collaborations and open research practices. Nonetheless, guidance on how these different commitments should be navigated and enacted is under‐defined.

The lack of guidance on how to enact commitments towards equity, responsibility, development and openness is compounded by traits within ethics discourse. Indeed, discussions around these topics remain largely distinct and lack explicit inter‐topic linkages.^30^ This an mean that different frameworks are used in response to different ethical issues, which can be both confusing and frustrating. The lack of guidance can be the cause of considerable ethical stress for researchers receiving these funds, particularly researchers from disciplines that do not have long‐standing traditions in engagement with (bio)medical ethics, capacity building, participatory research or LMIC/HIC collaboration. In order to unpack some of these ethical tensions we now turn to a case study project recently completed by some of the authors.

The Open Laboratory Instrumentation (OLI) is a recent GCRF‐funded project that was a collaboration between many of the authors on this paper. This collaboration involved physicists in the UK and engineers in Tanzania. In response to the high upfront costs and maintenance costs of commercially‐available research/clinical microscopes, the project aimed to develop “an open‐source, 3D‐printed, and fully‐automated laboratory microscope, with motorised sample positioning and focus control.”^31^ The resultant microscope is highly customisable, with a number of options readily available including trans‐ and epi‐ illumination, polarisation contrast imaging, and epi‐florescence imaging.

The OLI project aimed to enable low‐volume manufacturing and maintenance by local personnel using open source 3D printing, vastly increasing accessibility. This included work on the underpinning hardware and software for sharing and reproducing 3D printed designs, as well as development of the microscope. Over the course of the project, the team and their collaborators in Africa produced over 100 microscopes in Tanzania for educational, scientific, and clinical applications. This is an important demonstration of the viability of local manufacturing as a means of countering existing challenges of costly, slow and unreliable international supply chains.^32^

In order to achieve the goals of microscope design and sustainable distributed manufacturing, OLI was designed as an Open Hardware (OH) project. OH evolved from the Open Science movement and commits to using open research practices to (re)design physical technologies. OH designs (ie. mechanical drawings, schematics, bills of materials, source code etc), in addition to the software that drives the hardware, are all released under free/libre terms. This means that the technology can be shared, adapted and constructed by downstream users at no additional cost.

### 3.1 Ethics and the OLI

The innovative approach to microscopy design, the intention to establish manufacturing practices in a LMIC (Tanzania) and the commitment to OH meant that the OLI clearly met both RRI and GCRF objectives. Nonetheless, integrating these two key areas of ethical responsibility into the project was not without challenges. The researchers on the OLI project came from engineering and physics background and, as many GCRF recipients in these disciplines, had no prior experience conducting research with a development agenda. Moreover, while the UKRI promotes frameworks mediating technology research such as RRI, access to training in these frameworks varied. The experiences from the OLI project thus enables reflection on a number of different issues. First, it provides the opportunity to reflect on the challenges experienced by researchers who are expected to adopt a development‐focused research agenda without prior training in development activities. Second, it allows critical reflection on how RRI practices are implemented by researchers working on collaborative co‐design projects between HICs and LMICs. These are discussed in more detail in the following sections.

### 3.2 Addressing development commitments

The development agenda of the GCRF related to the OLI focus on digital diagnostic technology fabrication. In order to address this, the OLI project needed to demonstrate the feasibility of designing an OH, 3D‐printed microscope that could be fabricated to the same specifications and standards in both the UK and Tanzania (“proof‐of‐concept”). Nonetheless, to truly achieve long‐term sustainable development, the researchers needed to demonstrate that this fabrication could continue after the end of the GCRF funding (long‐term sustainability). As the GCRF funding was capped at 3 years, these two expectations meant that the researchers needed to reconcile the joint expectations of producing a working microscope and fostering long‐term development capacity building became topics of lengthy discussion.

### 3.3 Proof‐of‐concept

The OLI project linked directly to the development agenda of the GCRF through the potential to build capacity for digital diagnostic technology manufacturing in Tanzania. In order to realise this goal, the project needed not only to demonstrate proof‐of‐concept (ie. that this was feasible within a Tanzanian context), but also to illustrate how sustainable distributed manufacturing could be taken forward after the end of the project.

In order to demonstrate proof‐of‐concept for distributed manufacturing capabilities the researchers set up reliable and sustainable prototyping/manufacturing systems in two very different contexts–the UK and Tanzania.^33^ These activities foregrounded a range of challenges within the Tanzanian context that were less prominent in the UK context and were related to complicated supply chains for hardware acquisition. These included unreliable/delayed delivery, quality/cost compromises and high importing duties.

While issues of supply chains challenges were common to the Tanzanian collaborators, the UK collaborators who instigated the grant had little prior experience of research/work on the African continent. Recognizing the problems of designing equipment for a context that one had no understanding of led to the inclusion of reciprocal exchange visits between the two collaborating groups. This enabled researchers to gain a nuanced understanding of the supply chains and manufacturing constraints in their partner countries. The trips rapidly became fundamental for the project's success. All of the collaborators agreed that these visits improved the quality of the final product, as it evolved to suit the environment in which it was to be used.

Having all members of the collaboration understand issues such as fluctuations in internet and power availability was also key to effective communication. Similarly, understanding the availability of research tools and issues of procurement in different research contexts were key to setting realistic expectations. This contextual understanding was key to ensuring productive communication and proactive problem solving. Interestingly, it was not just the opportunity to visit the collaborating researchers that was important, but also the *length* of these visits. The OLI team members regularly spent up to a month at their collaboration sites, enabling them to get detailed understandings of daily routines and challenges. The long visits also fostered the establishment of close personal relationships, which were key to navigating communication throughout the project.

### 3.4 Longevity of project

The careful balancing act between openness, equity and pragmatism became apparent when the OLI team planned for the longevity of the project. While the openness of the technology remained a priority for post‐project plans, it was recognized that the long‐term production of the microscope in Tanzania would be a commercial venture. The challenge of building a self‐sustaining commercial venture producing open technologies in Tanzania were manifold, and required additional legal and administrative support to prepare the relevant documentation.

Nonetheless, the short length of most GCRF projects presented considerable challenges for the OLI team. The structure and administration of the grant meant that there was no funding for long‐term curation and refinement of the microscope design. The OLI team felt that they would have benefitted from more time to ensure that the project was working and that the technology could be sustainably manufactured after the end of the project. This linked directly into their interpretation of their development responsibilities: guaranteeing that the device can be working for six months at low cost.

In particular, the practice of planning for project longevity sometimes came into conflict with opportunities to help out collaborators and speed up the project outcomes. During the OLI project the researchers were required to balance the short‐term proof‐of‐concept goals with the commitment to long‐term sustainability. This sometimes meant not taking advantage of obvious short‐term gains, such as couriering hardware from the UK to Tanzanian in order to avoid purchase/delivery delays. The researchers thus had to deal with the delays in their research so as to ensure that all design/manufacturing processes were sustainable within a Tanzanian context once the OLI project had finished. Sometimes it would seem that the good of the project and collaboration team ran into conflict with the longer‐term objectives of the technology. For instance, the “good of the project” could have been interpreted as the obligation to send resources to Tanzania to facilitate the completion of the research. In contrast, it became apparent that the longevity of the broader microscope project was better served by encouraging the Tanzanian participants to find local sources of materials or other solutions so as to build sustainable fabrication systems for when the UK partners were no longer involved.

### 3.5 Ethical concerns associated with achieving development commitments

The development focus of the GCRF grant scheme was taken very seriously by all researchers on the OLI project. Nonetheless, without specific guidance on how to undertake effective development activities, the researchers had to rely largely on their own interpretation of how to put these commitments into practice. Their learning experiences in this realm highlighted three key areas that gave rise to continual reflection and concern.

### 3.6 Understanding the context

The RRI framework foregrounds the importance of designing socially acceptable and contextually‐appropriate technologies.^34^ This is also of fundamental importance for the development agenda, as technologies need to be both embedded within, and appropriate for, local socio‐technical systems. The OLI project highlighted two distinct challenges relating to this contextuality imperative. The first challenge related to the structure of current funding systems. The rapid turn‐around of grant calls can mean that researchers commit to collaborations with peers in countries that they have not necessarily visited. This can mean that researchers are ill‐equipped to take into consideration the material, discursive and organizational dimensions of the different research contexts. This requires detailed knowledge of global and local actors and how they contribute to tensions in different contexts.

The OLI project took important steps towards addressing caveats in contextual knowledge within the research group by prioritizing long visits to the different research contexts. This enabled the researchers to develop an important contextual sensitivity that not only assisted with the inter‐personal aspects of the collaboration, but also contributed to the strength of the resultant technology design. Without these visits it is difficult to envision how socially responsive technology can be developed, or how the anticipatory obligations from the RRI model could have been addressed.

The second challenge related to how the process of socially‐responsive design is managed in two highly disparate social contexts so as to feed meaningfully into a single design. Hitherto studies on technology design have focused on development within a single context^35^ or on the processes whereby technology moves from one context to another. Few studies to date consider the practices of co‐design between HIC and LMIC and the dialogue between not only the researchers and their contributing communities.

The OLI project foregrounded the challenges of co‐designing a single item of technology in two highly disparate contexts. The researchers had to continually engage in discussions to arrive at compromises that would enable the smooth co‐development of the microscope. This could mean altering design decisions (such as whether to include mobile phones as image‐capturing devices) or purposely slowing down the speed of the project to ensure long‐term sustainability. Whether the current RRI literature provides sufficient guidance to researchers aiming to develop concurrent technological development in two highly disparate contexts remains questionable.

### 3.7 Ensuring project relevance and longevity within a specific timeframe

The OLI team interpreted successful development to include ensuring longevity of the microscope technology after the funding had finished. While the GCRF grant had provided them with time to develop the technology, there was no natural follow‐on grant that would enable them to address issues such as licensing and user engagement that would be key for the technology's longevity.^36^ Interestingly, this again foregrounds problems with the way that technology is framed within the GCRF call. The assumption that technology, once developed, will integrate into society and assist the development agenda significantly contrasts with more socio‐technical approaches.^37^

One of the key lessons from RRI case studies has been the need for long‐term engagement with the technology and user communities.^38^ The structure of current funding initiatives, such as the GCRF, can leave researchers uneasy in leaving technology projects unfinished or poorly situated due to funding constraints. In particular, the restrictions of grant time‐frame curtail engagement with user public and the setting up of sustainable systems to ensure longevity of project post‐funding.^39^

While the OLI project finished with a tested and working microscope design that could be fabricated in Tanzania, the timeframe of the project meant that the researchers had not had the opportunity to support the registration of the device or the upskilling of the private Tanzanian company (Bongo Tech and Research Labs) that would be taking forward the long‐term fabrication. This meant that the burden to ensure the long‐term sustainability of the OpenFlexure microscope rested with the Tanzanian team and was not funded by the original grant. Recognizing such challenges highlights the need for more creative funding to bridge the gap between research and innovation. More support is needed for researchers to address issues of longevity and design them into research project. As mentioned by Franzen et al, what is needed is that research capacity outcomes are equally valued as research outcomes.^40^

### 3.8 Engagement of the public

Key to current discussions on the ethics of technology is the engagement of the public in the research project. While many research teams coming from the natural sciences have the technical expertise to develop functional technology within short time‐span, few have prior experience conducting participatory‐action research (PAR). Such was the case for the OLI team, represented by engineers and physicists. Implementing PAR into research designs would have required funds for additional expertise or considerable time‐investments from the researchers themselves.

Fixed budgets and timeframes mean that there is often no time for extended community engagement and situational analyses prior to the start of the project. As is foregrounded in medical research,^41^ the establishment of effective trust relationships between communities and researchers takes time and considerable investment. It is not something that should ‐ or can ‐ be entered into lightly. Indeed, without expertise in PAR the researchers could have skewed their development by either relying on a small number of individuals or hearsay. They could also have conducted poor PAR by ‘extracting' views from research stakeholders, rather than engaging in dialogue.

Such observations necessarily raise considerable concerns for GCRF projects. Effective RRI requires community participation in order to consider context, anticipate impacts, reflect on purposes, engage inclusively, and act responsibly. How these communities–be they clinicians, educators or researchers‐can be effectively engaged in highly technical projects with short timeframes is challenging‐particularly so in countries with low science literacy and little tradition of citizen engagement with research.

### 3.9 Addressing capacity building goals

One of the key objectives of the GCRF fund is to “strengthen capacity for research, innovation and knowledge exchange in the UK and developing countries through partnership with excellent UK research and researchers.”^42^ This placed an expectation on the OLI project that it would contribute to research capacity building within Tanzania and contribute to knowledge exchange. The OLI team interpreted this commitment in three key ways, first to facilitate learning opportunities for early career researchers on the project, second to foreground equity within the collaboration and third to facilitate knowledge exchange through Open Research (OR) practices. Integrating these commitments into the project design led to instances of ethical concern.

### 3.10 Integrating Open Research practices into cross‐cultural collaborations

OR is becoming an important element within research. Many funders (including the UKRI) require the integration of OR activities in the research design.^43^ These open work practices can include prioritising Open Access publications, use of Free and Open Source Software, making research data open and using open version control platforms such as Git to share project activities. The commitment to OR practices was a core element of the OLI project design.

Discussions around OR are relatively well‐established in the UK, and researchers have access to considerable support to assist the integration of OR practices into their projects. In contrast, OR topics tend to be relatively new for many LMIC researchers, and there is little in the way of institutional support to assist them to integrate OR practices into their work.

The OLI project team thus had to navigate a number of complexities relating to the integration of OR practices into their research design. OR was not only a requirement of the GCRF funding, it was also an integral part of the project as an OH activity. Nonetheless, how openness is integrated into research can vary considerably, and there is considerable scholarship describing OR practices as a continuum rather than a check list.^44^ Recognizing this led to two key realisations, first that the researchers more familiar with OR should not simply impose “ways of doing” onto the rest of the collaboration team. Second, that there were many ways in which the same goals of openness could be achieved.

While open work practices remained an integral part of the OLI roll‐out, the enactment of openness became a site of dialogue and constant revision. It required a complicated process of negotiation and compromise that relied on dialogue, trust and empathy. As openness is a personal value, strategic research approach, a commercial strategy and a means of communicating with stakeholders, finding a middle‐ground approach can be difficult. Finding acceptable means of operationalizing openness in research is as much a practice in self‐discipline as in compromise.

### 3.11 Achieving equity in project hierarchies

The overall goal of the GCRF funding was to support equitable, collaborative blue‐skies research. Nonetheless, when submitting the OLI grant it became apparent that adding the Tanzanian colleagues as co‐investigators was problematic on the required submission platform as they were not associated with a research institution. While the Tanzanian team were equal partners in terms of research decisions, this bureaucratic issue meant that they were positioned as “sub‐contractors” in terms of the grant administration. Without significant effort from everyone involved in administering the OLI grant, this could have led to transparency issues in relation to budgeting and reporting. In addition, the structure of the project related to the bureaucracy involved in grant administration. This led to complications in making the payments to the Tanzanian colleagues, leaving the team with cash flow issues as they waited for the money to clear.

### 3.12 Ethical challenges associated with capacity building commitments

One of the key objectives of the GCRF funding was to “strengthen capacity for research, innovation and knowledge exchange in the UK and developing countries through partnership with excellent UK research and researchers.”^45^ The successful implementation of these capacity building commitments requires more than the sharing of funds and expertise. A growing body of literature has highlighted the complexities of equitable LMIC/HIC collaborations, drawing attention to key issues such as power asymmetries within collaboration teams,^46^ ethics dumping,^47^ and issues of authorship, data sharing and professional development.^48^ Navigating such issues is often compounded by a lack of training for the researchers involved, and the absence of dedicated funds and support to assist in efforts.

During the OLI project the researchers had to deal with a number of these issues throughout the project. These manifested through the design of the grant bureaucracy, variations in project priorities and expected outcomes and infrastructural differences.

### 3.13 Navigating value‐heterogeneity within the project team

Within ethics of technology debates considerable attention is paid to how core societal values are identified are translated into design and practice. Discussions around RRI, for instance, often make reference to the agenda‐setting negotiations between researchers and members of society necessary for identifying and prioritising these values.^49^

Fewer discussions, however, address value‐heterogeneity research teams. In so doing, much current ethics education and guidance frames research teams as value‐homogenous and does not provide detailed guidance on how to navigate disparate value systems, disagreements and conflicting regulatory landscapes within cross‐cultural research teams. This is likely linked to the strong UK/EU‐focus of current RRI discussions where research teams operate within a similar geographic location, under similar legislation and with a common cultural background.

Effective cross‐cultural dialogue is an area of extensive study, as are effective cross‐cultural/national collaborations.^50^ This literature foregrounds the importance of personal openness and engagement in relationship building as a means of ensuring meaningful dialogue. It is recognized that this is not only a continuous process, but also a personal commitment.^51^ This requires considerable support and investment to ensure that the inter‐personal relationships are in place before the start of the grant, rather than negotiated solely during the research process.

It must be recognized that even in situations where researcher values appear to align, misunderstandings can still arise. In the OLI case study, the collective commitment to OR practices led to many discussions and compromises about how to operationalise openness within the project. While value‐alignment challenges are starting to be discussed in relation to the GCRF, these tend to focus on differences between global priorities and local realities rather than differences in personal preferences. Grieve and Mitchell, for example, offer the example that “aspects of global priority such as human rights and what these encompass may conflict with the reality on the ground.”^52^

It would thus seem that navigating value differences within the collaboration team are crucial to effective RRI engagement. Nonetheless, effective communication skills training and conflict resolution, as key skills for maintaining value‐focused discussions are rarely offered to researchers.

### 3.14 Creating a working team and addressing power asymmetries

As the majority of ethics of technology discussions have focused on HICs, project and research infrastructures are rarely foregrounded as topics of conversation. Indeed, the provision of project administration as well institutional and national infrastructures supporting research are taken as a *de facto* given. In contrast, GCRF funding is explicitly intended for collaborations with countries on the OECD Development Assistance Committee (DAC) list. It recognizes that “these countries often have the weakest and most fragile institutions, and limited resources to support research.”^53^

All the members of the OLI team mentioned the challenges of conducting research in Tanzania. These included infrastructural issues such as the delivery of project materials and lack of reliable power and internet. As the OLI team had spent considerable time developing interpersonal relationships, they were able navigate these issues constructively. Nonetheless, these situations continued to present instances of personal and moral unease. All members of the OLI team agreed that the instigation of regular meetings was vital in navigating these issues. Without these meetings, as well as the situational empathy developed through on‐site visits, it is possible that these resource asymmetries could have evolved into power asymmetries that impacted on the efficacy of the collaboration.

Appreciating the infrastructural inequality between collaborators in LMICs and HICs and successfully navigating these issues in a sensitive manner is rapidly becoming a “take home” message of the GCRF funding scheme.^54^ While such issues have long been discussed in relation to research capacity development,^55^ there was a marked lack of training for researchers funded on the scheme. Similarly, current RRI discussions tend to focus on power asymmetries between the research team and user‐public, or issues of gender and inclusion. They do not necessarily focus on power asymmetries due to different working environments.

The experiences of the OLI team foreground the need for better training in relationship management ‐ both in terms of GCRF funding and RRI. There is a need to support lead researchers in team building activities that avoid imposition of values and assumptions. In situations of infrastructural asymmetry nothing can be taken for granted, and one should not assume that communication is optimal between team members.

### 3.15 Differing narratives and priorities

The OLI project team represents an interesting challenge to current RRI discussions due to the differing priorities of the two national teams. While the UK team was primarily focused on research, the Tanzanian team was focused on the commercialization of the microscope. Thus, within the same project team *research* and *innovation* was variously prioritized amongst individuals. Luckily, these interests were not exclusionary, and the team were willing to engage with activities outside of their priority area.

Nonetheless, the existence of these differing priorities within the same project team foregrounds a challenge to RRI discussions. While it is tempting to think of differing end‐point priorities being negotiated between different stakeholders (ie. researchers and commercial companies), there is less guidance on how these negotiations occur within research teams.

A further challenge to navigating differing priorities is the role that technology plays in the narratives of the GCRF and RRI. The GCRF takes a generally positive view of technology as an enabler of development. In contrast, RRI discussions tend to take a precautionary approach and focus on ameliorating the negative impacts of sensitive technologies. Both, it would seem, tend to overlook the unequal distribution of the benefits of innovation and more contextual rich understandings of the flow of information through societies.

### 3.16 Addressing funding structures and underlying grant frameworks

While the GCRF funds are intended to support equitable collaborations between the UK and DAC countries, the funding scheme is located in the UK. The awarding, administration and reporting of these grants thus follows the UKRI model. This includes specific requirements regarding grant administration, finance and ethics approval that the DAC collaborators are expected to follow.

These expectations can place considerable strain on the research collaboration. As was evidenced by the discussions within the OLI team, these expectations were often difficult to implement. The Tanzanian collaborators, for example, had to rapidly adapt to the required methods of expense reporting, while the UK collaborators had to act as a mediator between the project team and their university finance department. In such cases, it needs to be recognized that these structures are imposed on the DAC researchers as a non‐negotiable aspect of the grant, and may not always be the priorities of those accepting the grant. Indeed, for many LMIC academics these represent a heavy burden as they attempt to align these expectations with their institutional bureaucracy that may not be equipped to provide them with the support they need.

Navigating such situations requires considerable commitment by all researchers, and a willingness to adapt and support each other so as to find an effective way forward. It is important to note that this success, in part, relies on the dispositions of the individual researchers exemplified by virtues such as humility, openness and curiosity. Such virtues are difficult to quantify, and definitely not listed on CVs or grant applications. Nonetheless, they were vital components in at least partially mitigating the inherent power imbalances between researchers in HIC and LMIC. It is possible to suggest that a group with different personalities or a different management style could have been much less successful by taking more traditional deficit approach that emphasized what the LMIC partners could not do compared to the HIC partners.

The challenges of identifying shared values in research and the imposition of bureaucratic and ethical structures or expectations on LMIC collaborators have been discussed in other areas.^56^ It is recognized that this requires a careful and thoughtful dialogue that takes into account possible tensions such as issues of scale or power imbalances. This presents interesting opportunities to discuss what constitutes responsible research management. Such topics are often intertwined with academic and funder bureaucracy,^57^ and how these are best understood across highly disparate contexts remains an area to be unpacked.

Similarly, the successful implementation of responsible research activities relies on more than the goodwill of the researchers. It requires in‐country oversight, ethics approval and expertise, all of which can be problematic for researchers working in LMICs, challenges that medical social sciences research has already documented.^58^ RRI implementation combines technical and ethical expertise and often falls out of the remit of ethics review boards, lack of institutional expertise in interdisciplinary research activities, and infrequent inclusion of social scientists on projects to address certain elements of RRI implementation.

## 4 CONCLUDING COMMENTS

The rapid expansion of health technology development continues to revolutionize healthcare provision. This impact is both global and local, and offers considerable potential for LMIC healthcare to advance through the ability to connect, rationalise and enrich existing health systems. Moreover, the potential for *in situ* design of contextually‐appropriate technologies offers an unprecedented level of control and agency for LMIC health systems.

International funding, such as the GCRF scheme, have been hugely influential in supporting health innovations in, and for, LMICs. These funding schemes, however, are not without challenges. The experiences of the OLI project highlight important ethical challenges that range from the structure of the grants to the design of contextually‐appropriate technologies. What is apparent from the discussion above is that these different areas of ethical concerns are currently covered by a range of different frameworks and/or fields of discourse. These include Open Science, development agendas, RRI and equitable partnership. While these different areas are, of course, linked by common values such as fairness, honesty and beneficence, the positioning and framing of these common values differs between these areas. Moreover, unified discussions that demonstrate how these areas are simultaneously enacted within a research context are lacking. This can leave researchers not only confused about how to respond to the differing commitments implicit in their grant, but worried that they are continually failing to live up to expectations. This realisation is particularly concerning when considering researchers who have no prior experience in, or training for, conducting research within these different frameworks.

Nonetheless, these challenges are not insurmountable. Indeed, the need to enrich ethical guidance around the development of LMIC‐appropriate health technologies can–at least in part–be done by linking together current areas of ethics scholarship. In particular, bringing these different areas of scholarship together to discuss these issues would be of considerable importance. For instance, the OLI team difficulties experienced in identifying OR practices that could be implemented by scholars in different research contexts with differing levels of computer literacy. Such issues could be offset by linking scholars on OR with development/capacity building scholars well‐versed in LMIC/HIC collaborative partnerships. Similarly, the difficulties of engaging the public in the OLI project could be offset by linking RRI scholars to ethicists specialising in community engagement and long‐term public‐academic relationships.

What becomes additionally apparent from the OLI project is the urgent need to integrate health technology research into current health research/ethics dialogues. As many researchers working on health technology projects come from disciplines not traditionally engaged in medical ethics discourse there is the potential that the wealth of expertise in this area will remain under‐utilized. In particular, key topics such as power dynamics/hierarchies in collaboration, LMIC/HIC collaborations, long‐term participant and community engagement are topics that would enrich and support health technology researchers.

Collaborations foreground issues of research environment asymmetry and resource allocation, infrastructural challenges and societal engagement that require careful consideration. This opens the potential for researchers in the HIC who have all the money, resources and taken‐for‐granted infrastructure to set the terms, expectations and ways of doing things and impose them on the LMIC team without discussion, perhaps buying into the western ‘rescue’ narrative towards LMICs and Africa in particular.

In addition to these challenges, the OLI project highlights the need for an enrichment of current RRI discourse so as to provide guidance for researchers designing technologies for use in LMIC contexts. Some of the issues raised by the OLI project, such as sustainability after funding, integration into LMIC socio‐technical systems, and public engagement, foreground the need for more thought on applying RRI in highly disparate contexts. They also highlight the critical need for training around managing research versus innovation expectations–both within the project team and beyond. These are issues that need to be further addressed by ethics communiteis around the world.

Linking RRI discussions to the current innovation ecosystems within LMICs, as well as the considerable expertise of NGOs and development specialists would not only enrich discussion, but support researchers with these aspects of their projects.^59^ Recognizing the enormity of this field will allow researchers not only a good idea of positive actions, but also a level of humility about what they are likely to achieve. As highlighted by Franzen et al, research capacity building outcomes need to be equally valued with research outcomes.^60^

The experiences from the OLI project provide much scope for reflection on research funding schemes for medical technologies that include capacity‐building expectations. The roll‐out of the project relied on the successful navigation of complex power‐dynamics arising from both the locus of the funding and the research contexts in which the activities occurred. It highlights the need for the challenges relating to capacity‐building activities to be more rigorously discussed and supported. Relying solely on individual researchers with no prior experience of capacity‐building activities is inappropriate and could undermine both research and development agendas. Indeed, shifting responsibility for mitigating research neo‐colonialism onto researchers undermines both the efficacy of the research‐as‐development agenda and brings into question the extent to which decolonization is a priority of funding councils and the governments that fund them.

In response to these challenges, there is growing global recognition of the need for governance and for educating researchers and innovators to responsibly produce technology that benefits society. Issues of participatory research and capacity building are brought to the fore in projects aimed at developing usable technology for LMICs. There is a fine line to navigate between cutting‐edge research and “tech dumping” by producing technology unsuited for LMIC contexts. Nor is it appropriate to place the entirety of the responsibility on the HIC researcher to ensure that the resultant technology is sustainable *in situ* after the close of the project. What is apparent, though, is the urgent need to find a workable balance to avoid the neo‐colonialization of technology research that it could unwittingly reproduce or reinforce relations of dependence between HICs and LMICs. Funders and the institutions that receive funding need to make better provision for training to ensure that researchers are equipped to deal with the complexities of these problems.

The recognition that ethical frameworks travel and evolve between research contexts is an important message for both GCRF and RRI discussions. In HIC‐LMIC collaborations the most responsible practice may sometimes be less about enforcing practices informed by ethical expectations, and more about being able to relinquish control. However, if research and innovation are understood as collective activities with uncertain and unpredictable consequences,^61^ how best control is negotiated requires far more discussion.

## FUNDING

OLI was funded by the Engineering and Physical Sciences Research Council (EPSRC) (ref. EP/P029426/1). Richard W. Bowman's salary was mostly paid by a Royal Society Fellowship (URF\R1\180153). The training course on “working across cultural divides” was supported by another Royal Society award (RGF\EA\181034). Julian Stirling was supported by the EPSRC (ref. EP/R013969/1).

## DECLARATION OF INTEREST STATEMENT

STICLab/Bongo Tech and Research Labs is a private company in Tanzania and is currently selling OpenFlexure Microscopes.

## Footnotes

1 Davis, N. (2016, January 19). What is the fourth industrial revolution? World Economic Forum. Retrieved November 29, 2021, from https://www.weforum.org/agenda/2016/01/what-is-the-fourth-industrial-revolution/

2 Maynard, A. D. (2015). Navigating the fourth industrial revolution. *Nature Nanotechnology*. *10*, 1005‐1006; Petrillo, A., De Felice, F., Cioffi, R., & Zomparelli, R. (2018). Fourth industrial revolution: Current practices, challenges, and opportunities. In A. Petrillo, R. Cioffi, & F. de Felice (Eds.), *Digital transformation in smart manufacturing*. London: IntechOpen.

3 Gleason, N. W. (Ed.). (2018). Higher education in the era of the fourth industrial revolution. Singapore: Palgrave Macmillan. pp. 1.

4 Von Schomberg, R. (2011). Prospects for technology assessment in a framework of responsible research and innovation. In M. Dusseldorp & R. Beecroft (Eds.), *Technikfolgen abschätzen lehren: Bildungspotenziale transdisziplinärer methoden*. Wiesbaden: Vs Verlag. pp. 40.

5 European Commission. (2020). Horizon 2020. Retrieved November 29, 2021, from https://ec.europa.eu/programmes/horizon2020/en/h2020-section/responsible-research-innovation

6 Stilgoe, J., Owen, R., & Macnaghten, P. (2013). Developing a framework for responsible innovation. *Research Policy*, *42*(9), 1568–1580.

7 UK Research and Innovation. (2021). Research integrity in healthcare technologies. Retrieved November 29, 2021, from https://epsrc.ukri.org/research/ourportfolio/themes/healthcaretechnologies/strategy/toolkit/home/integrity/ri/

8 Jakobsen, S., Fløysand, A., & Overton, J. (2019). Expanding the field of responsible research and innovation (RRI)–from responsible research to responsible innovation. *European Planning Studies*, *27*(12), 2329–2343.

9 Yaghmaei, E., & Van De Poel, I. (Eds.). (2021). Assessment of responsible innovation: Methods and practices. London: Routledge.

10 Macnaghten P., Owen R., Stilgoe J., Wynne B., Azevedo A., de Campos A., Chilvers J., Dagnino R., di Giulio G., Frow E., Garvey B., Groves C., Hartley S., Knobel M., Kobayashi E., Lehtonen M., Lezaun J., Mello L., Monteiro M., … Velho L. (2014). Responsible innovation across borders: Tensions, paradoxes and possibilities. *Journal of Responsible Innovation*, *1*(2), 191–199.

11 Wong, P. (2016). Responsible innovation for decent nonliberal peoples: A dilemma? *Journal of Responsible Innovation*, *3*(2), 154–168.

12 Hartley, S., McLeod, C., Clifford, M., Jewitt, S., & Ray, C. (2019). A retrospective analysis of responsible innovation for low‐technology innovation in the global south. *Journal of Responsible Innovation*, *6*(2), 143–162.

13 Macnaghten, Owen, Stilgoe, Wynne, Azevedo, de Campos, Chilvers, et al., op. cit. note 10, pp. 191.

14 Ibid: 191.

15 Ibid: 191.

16 This is a term used to describe the practice of researchers from high‐income countries who collect specimens/data from low/middle‐income countries yet conduct all the analysis and subsequent storage out of the country of origin.

17 Beran, D., Byass, P., Gbakima, A., Kahn, K., Sankoh, O., Tollman, S., Witham, M., & Davies, J. (2017). Research capacity building—Obligations for global health partners. *The Lancet Global Health*, *5*(6), e567‐e568; Khanal, P. (2017). Bringing all together for research capacity building in LMICs. *The Lancet Global Health*, *5*(9), e868.

18 Liu, A. (2019, September 1). Africa's future is innovation rather than industrialization. World economic forum. Retrieved November 29, 2021, from https://www.weforum.org/agenda/2019/09/africa-innovation-rather-than-industrialization/

19 United Nations. (2015). The 17 goals. Retrieved November 29, 2021, from https://sdgs.un.org/goals

20 Manji, A., & Mandler, P. (2019). Parliamentary scrutiny of aid spending: The case of the global challenges research fund. *Parliamentary Affairs*, *72*(2), 331–352.

21 Grieve, T., & Mitchell, R. (2020). Promoting meaningful and equitable relationships? Exploring the UK's global challenges research fund (GCRF) funding criteria from the perspectives of african partners. *The European Journal of Development Research*, *32*, 514–528.

22 Zaman, M., Afridi, H., Ohly, H., McArdle, H., & Lowe, N. (2020). Equitable partnerships in global health research. *Nature Food*, *1*(12), 760–761.

23 UK Strategy for the Global Challenges Research Fund (GCRF). (2016). Science and research funding allocation: 2016 to 2020. pp. 1. Retrieved November 29, 2021, from www.gov.uk/government/publications/science-and-research-funding-allocation-2016-to-2020

24 UK Research and Innovation. (2021). Global challenges research fund. Retrieved November 29, 2021, from https://www.ukri.org/research/global-challenges-research-fund/

25 Ibid.

26 Datta, A. (2018). Doing research for (and not on) development: Some important questions for the global challenges research fund. LSE impact blog. Retrieved November 29, 2021, from http://blogs.lse.ac.uk/impactofsocialsciences/2018/09/03/doing-research-for-and-not-on-development-some-important-questions-for-the-global-challenges-research-fund/

27 Ibid.

28 Larkan, F., Uduma, O., Lawal, S., & van Bavel, B. (2016). Developing a framework for successful research partnerships in global health. *Globalization and Health*, *12*(1), 1–9; Schroeder, D., Chatfield, K., Singh, M., Chennells, R., & Herissone‐Kelly, P. (2019). Equitable research partnerships: A global code of conduct to counter ethics dumping. Dordrecht: Springer; Zaman, Afridi, Ohly, McArdle, & Lowe, op. cit. note 22.

29 UK Strategy for the Global Challenges Research Fund, op. cit. note 23, pp. 8.

30 Thapa, R. K., Iakovleva, T., & Foss, L. (2019). Responsible research and innovation: A systematic review of the literature and its applications to regional studies. *European Planning Studies*, *27*(12), 2470–2490.

31 Collins J. T., Knapper J., Stirling J., Mduda J., Mkindi C., Mayagaya V., Mwakajinga G. A., Nyakyi P. T., Sanga V. L., Carbery D., White L., Dale S., Lim Z. J., Baumberg J. J., Cicuta P., McDermott S., Vodenicharski B. & Bowman R. (2020). Robotic microscopy for everyone: The OpenFlexure microscope. *Biomedical Optics Express*, *11*(5), 2447.

32 Ibid.

33 Ibid.

34 Stilgoe, Owen, & Macnaghten, op. cit. note 6.

35 Bijker, E., Parke Hughes, T., & Pinch, T. J. (2012). The social construction of technological systems: New directions in the sociology and history of technology. Cambridge, MA: MIT Press.

36 The team did secure an Impact Acceleration Award which provided some further funds.

37 Webster, A., & Gardner, J. (2019). Technology analysis & strategic management aligning technology and institutional readiness: The adoption of innovation. *Technology Analysis and Strategic Management*, *31*(10), 1229–1241.

38 van de Poel, I., Asveld, L., Flipse, S., Klaassen, P., Scholten, V., & Yaghmaei, E. (2017). Company strategies for responsible research and innovation (RRI): A conceptual model. *Sustainability*, *9*(11), 2045.

39 Morrison, M., Mourby, M., Gowans, H., Coy, S., & Kaye, J. (2020). Governance of research consortia: Challenges of implementing responsible research and innovation within Europe. *Life Sciences, Society and Policy*, *16*(1), 13.

40 Franzen, S. R. P., Chandler, C., & Lang, T. (2017). Health research capacity development in low and middle income countries: Reality or rhetoric? A systematic meta‐narrative review of the qualitative literature. *BMJ Open*, *7*(1), e012332.

41 Kerasidou, A. (2019). The role of trust in global health research collaborations. *Bioethics*, *33*(4), 495–501.

42 UK Research and Innovation, op. cit. note 24.

43 UK Research and Innovation. (2021). Open Research. Retrieved November 29, 2021, from https://www.ukri.org/our-work/supporting-healthy-research-and-innovation-culture/open-research/

44 Higman, R., Bangert, D., & Jones, S. (2019). Three camps, one destination: The intersections of research data management, FAIR and open. *Insights: the UKSG Journal*, *32*(1), 18.

45 UK Research and Innovation, op. cit. note 24.

46 Macnaghten, Owen, Stilgoe, Wynne, Azevedo, de Campos, Chilvers, et al., op. cit. note 10.

47 Schroeder, D., Cook, J., Hirsch, F., Fenet, S., & Muthuswamy, V. (2018). Ethics dumping case studies from north‐south research collaborations. In D. Schroeder, J. Cook, F. Hirsch, S. Fenet, & V. Muthuswamy (Eds.), Springer briefs in research and innovation governance. Dordrecht: Springer International Publishing.

48 Smith, E., Hunt, M., & Master, Z. (2014). Authorship ethics in global health research partnerships between researchers from low or middle income countries and high income countries. *BMC Medical Ethics*, *15*(1), 1–8.

49 Stilgoe, Owen, & Macnaghten, op. cit. note 6.

50 Gudykunst, W. B. (2003). Cross‐cultural and intercultural communication. Amsterdam: SAGE Publications; Bowe, H., Martin, K., & Manns, H. (2014). Communication across cultures: Mutual understanding in a global world. Cambridge: Cambridge University Press.

51 The UK team did participate in a training day on working across cultural divides, that they found was a great opportunity to reflect and improve their practice. Nonetheless, continuous support for these activities was lacking.

52 Grieve, & Mitchell, op. cit. note 21.

53 UK Strategy for the Global Challenges Research Fund, op. cit. note 23, pp. 7.

54 Grieve & Mitchell, op. cit. note 21, pp. 523.

55 Kasprowicz V. O., Chopera D., Waddilove K. D., Brockman M. A., Gilmour J., Hunter E., Kilembe W., Karita E., Gaseitsiwe S., Sanders E. J., & Ndung'u T. (2020). African‐led health research and capacity building‐ Is it working? *BMC Public Health*, 20, 1104.

56 Bezuidenhout, L. (2014). Ethics in the minutiae: Examining the role of the physical laboratory pnvironment in ethical discourse. *Science and Engineering Ethics*, *21*, 51–73; Schroeder, Cook, Hirsch, Fenet, & Muthuswamy, op. cit. note 47.

57 Morrison,, Mourby, Gowans, Coy, & Kaye, op. cit. note 39.

58 Franzen, Chandler, & Lang, op. cit. note 40.

59 The OLI project collaborated with UK NGOs “Tech for Trade” and “The Centre for Global Equality” to assist with the development‐related aspects of their work.

60 Franzen, Chandler, & Lang, op. cit. note 40.

61 Owen, R., Macnaughten, P., & Stilgoe, J. (2012). Responsible research and innovation: From science in society to science for society, with society. *Science and Public Policy*, *39*(6), 751–760.

